# Competitive formation between 2D and 3D metal-organic frameworks: insights into the selective formation and lamination of a 2D MOF

**DOI:** 10.1107/S2052252519007760

**Published:** 2019-06-12

**Authors:** Sojin Oh, Jeehyun Park, Moonhyun Oh

**Affiliations:** a Department of Chemistry, Yonsei University, 50 Yonsei-ro, Seodaemun-gu, Seoul 03722, Republic of Korea

**Keywords:** metal-organic frameworks, 2D layered MOFs, structural control, lamination

## Abstract

The structural dimension of metal–organic frameworks (MOFs) is of great importance in defining their properties and thus applications. In particular, 2D layered MOFs are of considerable interest because of their useful applications, which are facilitated by unique structural features of 2D materials, such as a large number of open active sites and high surface areas. Herein, this work demonstrates a methodology for the selective synthesis of a 2D layered MOF in the presence of the competitive formation of a 3D MOF. The ratio of the reactants, metal ions and organic building blocks used during the reaction is found to be critical for the selective formation of a 2D MOF, and is associated with its chemical composition. In addition, the well defined and uniform micro-sized 2D MOF particles are successfully synthesized in the presence of an ultrasonic dispersion. Moreover, the laminated 2D MOF layers are directly synthesized via a modified bottom-up lamination method, a combination of chemical and physical stimuli, in the presence of surfactant and ultrasonication.

## Abstract 2

A method for the selective synthesis of a 2D layered MOF in the presence of the competitive formation of a 3D cubic MOF was developed. In addition, the laminated 2D MOF layers are directly synthesized via a modified bottom-up lamination method involving both chemical (surfactants) and physical (ultrasonication) stimuli.

## 1. Introduction   

Metal–organic frameworks (MOFs) are an interesting class of well ordered crystalline materials that can be constructed from many combinations of multitopic organic ligands and metal ions or clusters. MOFs have received a great deal of attention because of their many useful properties and potential in various applications such as gas storage, separation, sensing and catalysis (Bohnsack *et al.*, 2013 ▸; Xie *et al.*, 2018 ▸; Sen *et al.*, 2017 ▸; Flaig *et al.*, 2017 ▸; Cho *et al.*, 2014*b* ▸; Xu *et al.*, 2017 ▸; Yi *et al.*, 2016 ▸; Chen *et al.*, 2017 ▸; Jiao *et al.*, 2017 ▸; Yoon *et al.*, 2019 ▸). In particular, the structure of a MOF is the most important factor in determining its properties and applications (Lu *et al.*, 2014 ▸; Cho *et al.*, 2014*a* ▸; Lee *et al.*, 2009 ▸; Ji *et al.*, 2018 ▸; An *et al.*, 2010 ▸). Additionally, size and morphology controls of MOFs are necessary for their finest utilization in many applications (Gao *et al.*, 2018 ▸; Wang *et al.*, 2018 ▸; Pham *et al.*, 2012 ▸). Recently, 2D layered MOFs have received considerable attention because of their unique features, such as many open active sites and high surface areas, making them optimal for use in catalysis, sensing and separation (Duan *et al.*, 2017 ▸; He *et al.*, 2018 ▸; Campbell *et al.*, 2015 ▸; Wang *et al.*, 2016 ▸; Peng *et al.*, 2017 ▸; Rodenas *et al.*, 2015 ▸). Thus, a selective synthesis of a 2D MOF against a 3D MOF is of great interest in MOF development. In addition, the preparation of laminated 2D MOF layers with a high aspect ratio is very important because the open active sites and surface area of 2D MOFs is higher as laminated 2D MOF layers (Zhao *et al.*, 2017 ▸, 2015 ▸, 2016 ▸; Cao *et al.*, 2016 ▸; Peng *et al.*, 2014 ▸; Hermosa *et al.*, 2015 ▸; Ding *et al.*, 2017 ▸; Foster *et al.*, 2016 ▸; Junggeburth *et al.*, 2013 ▸; Xue *et al.*, 2018 ▸; Zhang *et al.*, 2019 ▸; Guo *et al.*, 2018 ▸). In general, two methods, a top-down and a bottom-up, have been developed for the preparation of laminated 2D MOF layers with a high aspect ratio. The top-down method is an indirect synthetic method, and is normally accompanied by a post-treatment process of bulk (stacked) 2D MOFs to laminate MOF layers using ultrasonication, ball-milling or intercalation (Peng *et al.*, 2014 ▸; Hermosa *et al.*, 2015 ▸; Ding *et al.*, 2017 ▸; Foster *et al.*, 2016 ▸). For example, Peng *et al.* (2014 ▸) developed a soft-physical lamination method involving a wet ball-milling process followed by ultrasonication of the bulk 2D MOF for the formation of laminated MOF layers (Peng *et al.*, 2014 ▸). In contrast, the bottom-up method is a direct one-pot synthetic method using a chemical stimulus such as surfactant molecules, which act as interrupters in the stacking process of the 2D MOF layers during the MOF formation (Zhao *et al.*, 2015 ▸; Junggeburth *et al.*, 2013 ▸; Xue *et al.*, 2018 ▸). For example, Zhao *et al.* (2015 ▸) developed a surfactant-assisted direct synthetic method for the preparation of ultrathin 2D MOF nanosheets (Zhao *et al.*, 2015 ▸).

Some 2D and 3D MOFs with similar chemical compositions can be constructed from identical metal ions and organic building blocks. For instance, the 3D ZIF known as ZIF-8 (Park *et al.*, 2006 ▸; Park & Oh, 2017 ▸), with a chemical composition of [Zn(MeIm)_2_]_*n*_, and 2D ZIF (Chen *et al.*, 2013 ▸) with a chemical composition of [Zn(MeIm)_2_(HMeIm)_1/2_(H_2_O)_3/2_]_*n*_ can be synthesized from identical reactants: Zn^2+^ and 2-methyl­imidazole (HMeIm). In addition, a well known 3D MOF known as MOF-5 (Li *et al.*, 1999 ▸; Son *et al.*, 2008 ▸) [Zn_4_O(BDC)_3_]_*n*_, and a 2D MOF with a chemical composition of [Zn_3_(BDC)_3_(DMA)_2_]_*n*_ (Wang *et al.*, 2008 ▸) can be constructed from Zn^2+^ and 1,4-benzene­dicarb­oxy­lic acid (H_2_BDC). However, no clear understanding on the selective construction of 2D versus 3D MOFs from identical building blocks has been reported. Herein, we report the methodology for the selective synthesis of a 2D MOF in the presence of the competitive formation of a 3D MOF. The relative ratio between metal ions and organic building blocks used during the reaction was found to be a critical point for the selective formation of 2D and 3D MOFs, relating to their chemical compositions. In addition, we have found that a modified bottom-up lamination method, a one-step solvothermal reaction of reactants in the presence of both chemical (surfactant) and physical (ultrasonication) stimuli, can directly synthesize the laminated 2D MOF layers from molecular building blocks.

## 2. Experimental   

### 2.1. General methods   

All solvents and chemicals were obtained from commercial sources and used as-received. Scanning electron microscopy (SEM) images were acquired using a *JEOL* JSM-6701 F field-emission instrument and a Hitachi SU 1510 SEM. Transmission electron microscopy (TEM) images and the selected area electron diffraction (SAED) patterns were acquired using a *JEOL* JEM-2100 F at 200 kV (Center for Microcrystal Assembly, Sogang University). Atomic force microscopy (AFM) images were acquired using a Park NX10. The simulated SAED patterns were acquired using the ‘*SingleCrystal*’ interface (Version 1.3.0) of the *CrystalMaker* software (Version 2.2.0; CrystalMaker Software Ltd). X-ray diffraction (XRD) patterns were measured using a Rigaku Ultima IV instrument equipped with a graphite monochromated Cu *K*α radiation source (40 kV, 40 mA). Infrared (IR) spectra of solid samples were obtained using a Jasco FT/IR 4200 spectrometer and an attenuated total reflection module. Thermogravimetric analysis (TGA) measurements were conducted using a Shimadzu TGA-50 under a nitro­gen atmosphere at a heating rate of 5°C min^−1^. ^1^H NMR spectra were recorded using a Bruker Advance II 300 spectrometer (^1^H NMR, 300 MHz) with chemical shifts reported relative to residual deuterated solvent peaks.

### 2.2. Synthesis   

#### 2.2.1. Synthesis of 3D-C-MOF and 2D-L-MOF   

A precursor solution was prepared by mixing Zn(NO_3_)_2_·6H_2_O (0.54 mmol, 160.6 mg) and various amounts of 1,4-benzene­dicarb­oxy­lic acid (H_2_BDC, 0.18, 0.36, 0.54, 1.08 or 1.62 mmol, 29.9, 59.8, 89.7, 179.4 or 269.1 mg) in 20 ml of *N,N*-di­methyl­acetamide (DMA). The resulting mixture was tightly sealed and placed in an oil bath (130°C) for 40 min. After 40 min, the resulting particles were isolated and subsequently washed several times with DMA and methyl­ene chloride via centrifugation–redispersion cycles. Each successive supernatant was decanted and replaced with fresh solvent.

#### 2.2.2. Synthesis of rhombus particles of 2D-L-MOF   

A precursor solution was prepared by mixing Zn(NO_3_)_2_·6H_2_O (0.54 mmol) and H_2_BDC (1.62 mmol) in 20 ml of DMA. The resulting mixture was heated in an oil bath for 30 min (at 130°C) in the presence of a stirring process or heated in an oil bath for 30 min (at 100°C) with an ultrasonic dispersion. After 30 min, the resulting particles were isolated and subsequently washed several times with DMA and methyl­ene chloride via centrifugation–redispersion cycles. Each successive supernatant was decanted and replaced with fresh solvent.

#### 2.2.3. Synthesis of uniform rhombus particles of 2D-L-MOF   

A precursor solution was prepared by mixing Zn(NO_3_)_2_·6H_2_O (0.54 mmol) and H_2_BDC (1.62 mmol) in 20 ml of DMA. The resulting mixture was heated in an oil bath (100°C) with an ultrasonic dispersion. When the nucleation starts after 20 min, ultrasonic dispersion was halted and the resulting mixture was kept in an oil bath for an additional 3 min for maturation. Following this, the resultant particles were isolated and subsequently washed several times with DMA and methyl­ene chloride via centrifugation–redispersion cycles. Each successive supernatant was decanted and replaced with fresh solvent.

#### 2.2.4. Synthesis of laminated 2D-L-MOF disks   

Polyvinyl­pyrrolidone (PVP, 300 mg) was dissolved in DMA solution (10 ml) containing Zn(NO_3_)_2_·6H_2_O (0.54 mmol) under sonication for 10 min. Following this, a DMA solution (10 ml) of H_2_BDC (1.62 mmol) was added to this solution, and the resultant mixture was heated in an oil bath (100°C) for 1 h with an ultrasonic dispersion. After 1 h, the resultant particles were isolated and subsequently washed several times with DMA and methyl­ene chloride via centrifugation–redispersion cycles. Each successive supernatant was decanted and replaced with fresh solvent.

## 3. Results and discussion   

**Figure 1:**
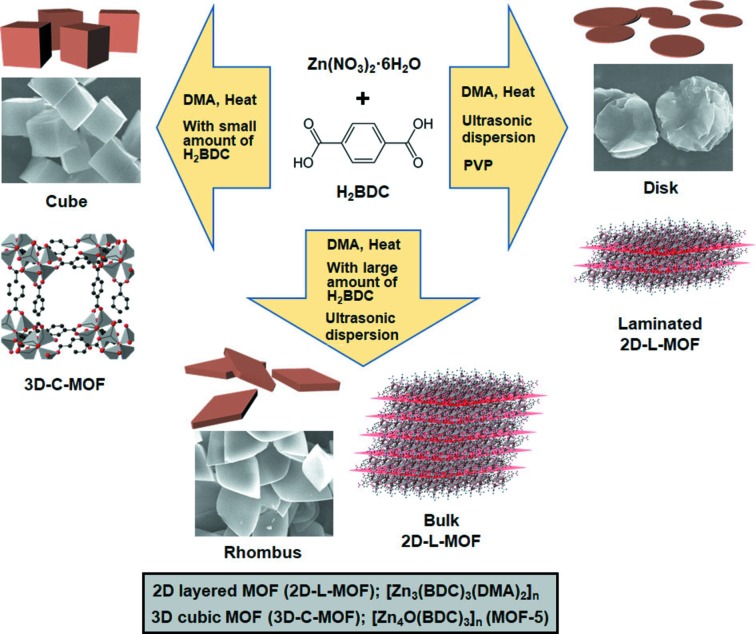
Selective formation of 2D and 3D MOF particles: 3D cubes, 2D rhombuses and 2D disks. All MOF particles were created from identical reactants, Zn(NO_3_)_2_ and H_2_BDC, but with different amounts and/or different synthetic conditions.

**Figure 2:**
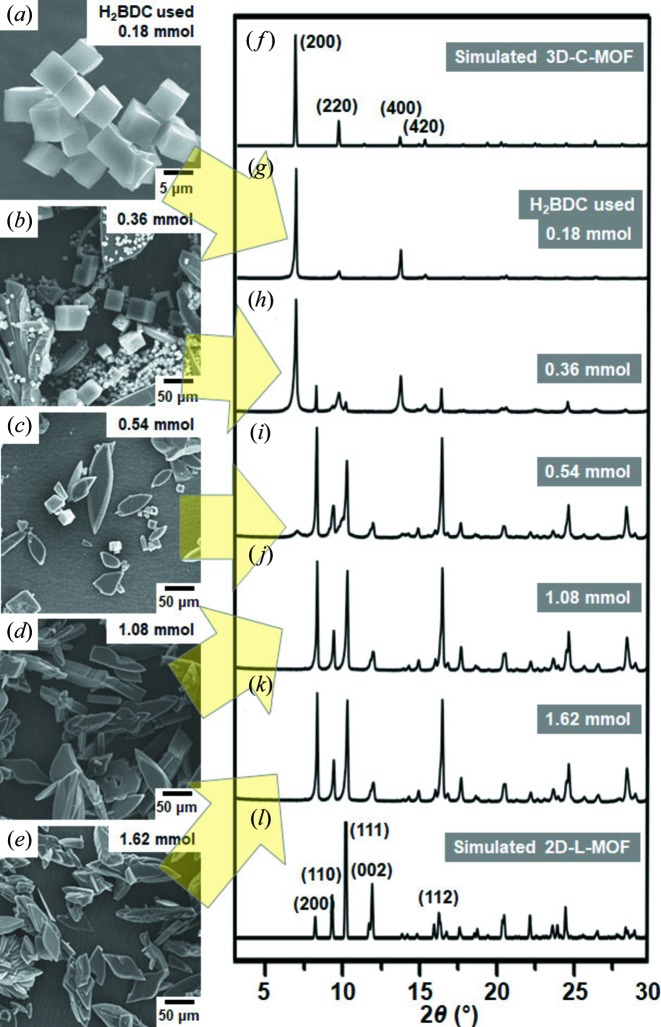
(*a*)–(*e*) SEM images showing the MOF particles produced from a series of solvothermal reactions in the presence of various amounts of H_2_BDC: (*a*) 0.18, (*b*) 0.36, (*c*) 0.54, (*d*) 1.08 and (*e*) 1.62 mmol, while maintaining the same amount of Zn(NO_3_)_2_ at 0.54 mmol. Simulated PXRD patterns of (*f*) 3D-C-MOF (MOF-5) and (*l*) 2D-L-MOF. (*g*)–(*k*) PXRD patterns of the MOF particles produced from a series of solvothermal reactions in the presence of various amounts of H_2_BDC: (*g*) 0.18, (*h*) 0.36, (*i*) 0.54, (*j*) 1.08 and (*k*) 1.62 mmol.

**Figure 3:**
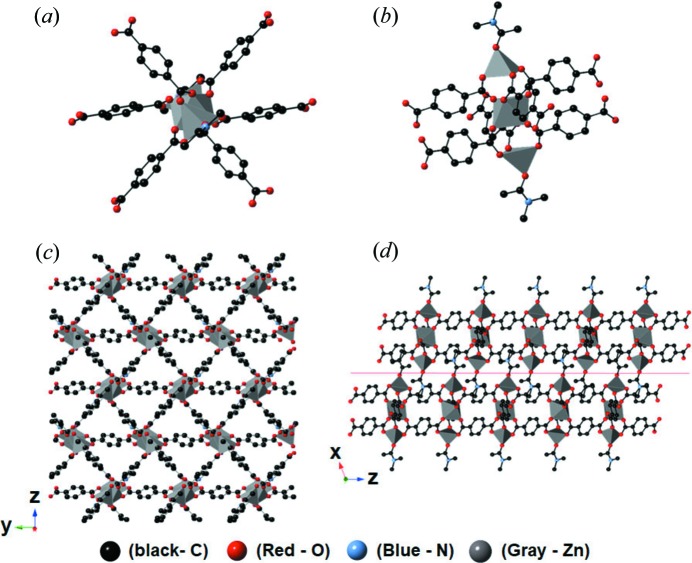
Ball-and-stick representations of 2D-L-MOF (Wang *et al.*, 2008 ▸). (*a*) and (*b*) Trimetallic building blocks of 2D-L-MOF, (*c*) one single layer of 2D-L-MOF and (*d*) two stacked layers of 2D-L-MOF. Hydrogen atoms have been omitted for clarity. Black, carbon; red, oxygen; blue, nitro­gen; gray, zinc.

It is very well known that the solvothermal reaction of Zn(NO_3_)_2_ (0.54 mmol) and H_2_BDC (0.18 mmol) in DMA results in the formation of cubic particles of 3D MOF (MOF-5) (Li *et al.*, 1999 ▸; Son *et al.*, 2008 ▸), which has a cubic structure and a chemical composition of [Zn_4_O(BDC)_3_]_*n*_ [hereafter denoted as 3D-C-MOF, see Figs. 1 ▸, 2 ▸(*a*) and 2 ▸(*g*)]. However, a similar solvothermal reaction of exactly the same reactants of Zn(NO_3_)_2_ and H_2_BDC only in the presence of an excess amount of H_2_BDC (1.62 mmol) resulted in large rhombus particles with some irregularly shaped particles [probably broken rhombuses, see Fig. 2 ▸(*e*)]. IR spectra of these rhombus particles indicated the coordination of the carboxyl­ate groups of BDC^2−^ to Zn^2+^, as showed by the characteristic shift of the CO stretching band from 1579.4 cm^−1^ (uncoordinated H_2_BDC) to 1673.4 cm^−1^ after the reaction (Fig. S1 in the Supporting Information). In addition, the powder X-ray diffraction (PXRD) pattern of these rhombus particles [Fig. 2 ▸(*k*)] was well matched to the simulated pattern of a reported 2D MOF [Fig. 2 ▸(*l*)], which has a layered structure with a chemical composition of [Zn_3_(BDC)_3_(DMA)_2_]_*n*_ (Wang *et al.*, 2008 ▸; see Fig. 3 ▸ for the detailed structure, hereafter denoted as 2D-L-MOF). Reviewing the amounts of the two reactants (Zn^2+^ and H_2_BDC) used during the reactions, 3D-C-MOF was favorably constructed from the reaction in the presence of a small amount of organic ligand (and thus a high metal-to-ligand ratio); however, 2D-L-MOF was preferentially produced from a similar reaction but in the presence of an excess amount of organic ligand (and thus a high ligand-to-metal ratio). On the basis of their compositions, [Zn_4_O(BDC)_3_]_*n*_ for 3D-C-MOF and [Zn_3_(BDC)_3_(DMA)_2_]_*n*_ for 2D-L-MOF, the metal-to-ligand (Zn^2+^-to-BDC^2-^) ratios incorporated within 3D-C-MOF and 2D-L-MOF were 4/3 and 3/3, respectively. The relative amount of ligand incorporated within 2D-L-MOF is larger than that incorporated within 3D-C-MOF. So more ligand per unit metal ion is required to construct 2D-L-MOF; therefore, the reaction conditions presenting a large amount of ligand in the reaction solution should be a favorable environment for the formation of 2D-L-MOF instead of 3D-C-MOF. Based on this assumption, we speculated that the high metal-to-ligand ratio of the reactants during the reaction may drive the formation of 3D-C-MOF rather than 2D-L-MOF because the Zn^2+^-to-BDC^2-^ ratio incorporated within 3D-C-MOF is larger than that in 2D-L-MOF. Using a similar approach, the formation of 2D-L-MOF rather than 3D-C-MOF is favorable in the reaction with the high ligand-to-metal ratio of reactants.

To validate this assumption, we conducted a series of solvothermal reactions in the presence of various amounts of H_2_BDC between 0.18 and 1.62 mmol (0.36, 0.54 and 1.08 mmol) while maintaining the amount of Zn(NO_3_)_2_ at 0.54 mmol. First, it should be noted that the cubic particles of 3D-C-MOF were exclusively generated from the reaction of Zn^2+^ (0.54 mmol) and a small amount of H_2_BDC (0.18 mmol). However, a mixture of small and large cubic particles as well as large rhombus and broken rhombus particles, which are the characteristic morphologies of 3D-C-MOF and 2D-L-MOF, respectively, was observed in SEM images [Fig. 2 ▸(*b*)] from the reaction of Zn^2+^ and H_2_BDC (0.36 mmol). Based on the morphology of the product, we temporarily concluded that a mixture of 3D-C-MOF and 2D-L-MOF had been formed. Indeed, the PXRD pattern of this product confirmed the formation of a mixture of 3D-C-MOF and 2D-L-MOF, as two characteristic sets of PXRD peaks corresponding to the 3D-C-MOF and 2D-L-MOF were detected [Fig. 2 ▸(*h*)]. On increasing the amount of H_2_BDC to 0.54 mmol, the formation of large rhombus and broken rhombus particles was dominant with a trace amount of cubic particles [Fig. 2 ▸(*c*)], and so the formation of 2D-L-MOF was predominant with a small portion of 3D-C-MOF. Indeed, only trace PXRD peaks representing 3D-C-MOF and the dominant PXRD peaks representing 2D-L-MOF were observed in the PXRD pattern [Fig. 2 ▸(*i*)]. Lastly, the reaction wherethe amount of H_2_BDC was increased to 1.08 mmol resulted in only large rhombus and broken rhombus particles without the formation of cubic particles, and thus possibly the selective formation of 2D-L-MOF without 3D-C-MOF [Fig. 2 ▸(*d*)]. Indeed, the characteristic PXRD peaks for 2D-L-MOF were only observed in the PXRD pattern [Fig. 2 ▸(*j*)]. In summary, either 3D-C-MOF or 2D-L-MOF were selectively synthesized in the presence of small or excess amounts of organic ligand, respectively, and a mixture of 3D-C-MOF and 2D-L-MOF was generated using ‘in-between’ amounts of organic ligand.

**Figure 4:**
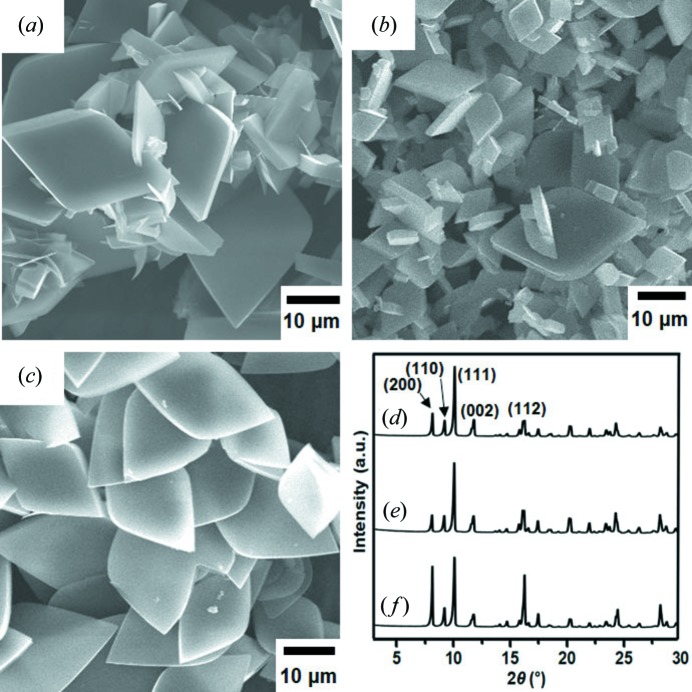
(*a*) SEM image of the rhombus 2D-L-MOF particles obtained from the solvothermal reaction of Zn(NO_3_)_2_ (0.54 mmol) and H_2_BDC (1.62 mmol) in the presence of a stirring process. (*b*) SEM image of the rhombus 2D-L-MOF particles obtained from the solvothermal reaction of Zn(NO_3_)_2_ (0.54 mmol) and H_2_BDC (1.62 mmol) in the presence of an ultrasonic dispersion process (solvothermal ultrasonic reactions). (*c*) SEM image of the rhombus 2D-L-MOF particles obtained from the solvothermal ultrasonic reaction of Zn(NO_3_)_2_ (0.54 mmol) and H_2_BDC (1.62 mmol); however, an ultrasonic dispersion process was stopped immediately after the initial seed formation. (*d*)–(*f*) PXRD patterns of the rhombus 2D-L-MOF particles shown in (*a*)–(*c*), respectively.

Thus far, we have concluded that 2D-L-MOF formed exclusively without the formation of 3D-C-MOF under the condition of an excess amount of H_2_BDC (high ligand-to-metal ratio). Subsequently, we attempted to produce more uniform particles of 2D-L-MOF with a regular shape and narrow size distribution. After numerous attempts, we discovered that the solvothermal reaction of Zn^2+^ (0.54 mmol) and H_2_BDC (1.62 mmol), only in the presence of a stirring process, resulted in the formation of regular rhombus particles but still with a broad size distribution [Fig. 4 ▸(*a*)]. The PXRD pattern of the rhombus particles showed that these particles are 2D-L-MOF [Fig. 4 ▸(*d*)]. To provide a similar but improved mixing effect, the solvothermal reaction of Zn^2+^ and H_2_BDC in the presence of an ultrasonic dispersion (hereafter denoted as a solvothermal ultrasonic reaction) was conducted and this reaction gave more regular and smaller rhombus particles [Fig. 4 ▸(*b*)]. This solvothermal ultrasonic reaction was then further modified to narrow the size distribution. Seeds of 2D-L-MOF may be generated at various points during the ultrasonic dispersion and thus the given growing times for individual seeds are different depending upon seed-formed time point. To prevent a delayed seed formation, the ultrasonic dispersion process was stopped at the time point of the initial seed formation (*ca*. 20 min) and the reaction was further matured during a short additional time (3 min) without ultrasonic dispersion. Indeed, we have succeeded in synthesising the uniform-sized rhombus particles without the small particles from a modified solvothermal ultrasonic reaction [Fig. 4 ▸(*c*)]. The PXRD pattern of these rhombus particles showed the exclusive formation of 2D-L-MOF without 3D-C-MOF [Fig. 4 ▸(*f*)].

**Figure 5:**
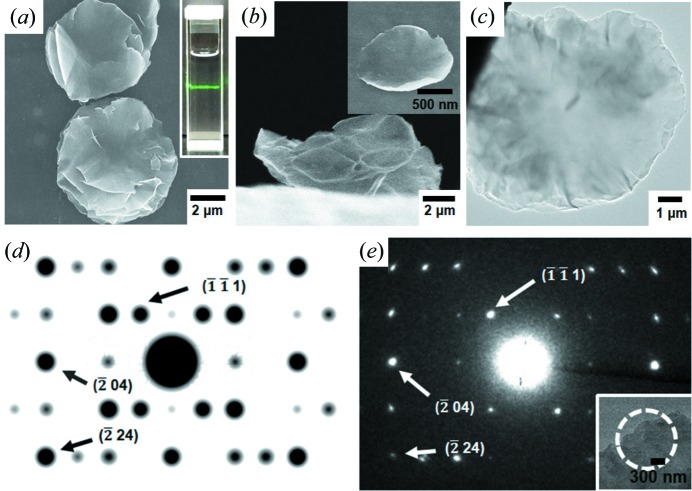
(*a*) and (*b*) SEM images showing the 2D-L-MOF disks obtained from the solvothermal ultrasonic reaction of Zn(NO_3_)_2_ (0.54 mmol) and H_2_BDC (1.62 mmol) in the presence of PVP. The inset in (*a*) shows a Tyndall effect of the colloidal suspension. (*c*) TEM image of a laminated disk of 2D-L-MOF. (*d*) Simulated SAED pattern of 2D-L-MOF. (*e*) Experimental SAED pattern of a laminated disk of 2D-L-MOF measured in the region marked with a circle in the TEM image (shown as an inset).

Some surfactant molecules, such as polyvinyl­pyrrolidone (PVP) and cetyltri­methyl­ammonium bromide (CTAB), can effectively interact with the surfaces of 2D materials, and thus they can interfere with the stacking of 2D layers, eventually resulting in laminated 2D layers (Zhao *et al.*, 2015 ▸; Junggeburth *et al.*, 2013 ▸; Xue *et al.*, 2018 ▸). On the basis of this phenomenon, we attempted to laminate the 2D-L-MOF layers using PVP. First, a surfactant-assisted method (chemical stimulus; see Fig. S2 for the resulting product from this method) or an ultrasonic dispersion process (physical stimulus) were unsuccessful in producing the laminated 2D-L-MOF layers. However, a modified bottom-up method, which combined both chemical and physical stimuli, resulted in the formation of the laminated 2D-L-MOF layers. The solvothermal reaction of Zn^2+^ and H_2_BDC was conducted in the presence of both PVP molecules and ultrasonic dispersion, and this reaction resulted in the formation of 2D disks with a relatively thin thickness instead of thick rhombus particles. Interestingly, we can observe partially laminated 2D-L-MOF layers and fully laminated 2D-L-MOF layers in the SEM images (Fig. 5 ▸). TEM images also showed the laminated 2D layer of 2D-L-MOF [Fig. 5 ▸(*c*)]. AFM images of the resulting 2D-L-MOF were also measured (Fig. S3); however, the thickness of samples and the precise information on the number of layers could not be obtained due to its waved morphology. SEM images of samples showing the waved morphology measured from side-view were also included (Fig. S4). The colloid suspension of the laminated 2D-L-MOF disks was clear but displayed a Tyndall effect upon irradiation using a laser [inset in Fig. 5 ▸(*a*)]. Although the morphology of the particles changed significantly from rhombus to disk, their PXRD pattern (Fig. S5) clearly showed that these disks are 2D-L-MOF. In addition, peak broadening was observed because of their thin-plate characteristic (Yang *et al.*, 2018 ▸; Pustovarenko *et al.*, 2018 ▸). Lastly, the SAED pattern measured from the laminated layer of 2D-L-MOF matched well with the simulated pattern of 2D-L-MOF [Figs. 5 ▸(*d*) and 5 ▸(*e*)].

**Figure 6:**
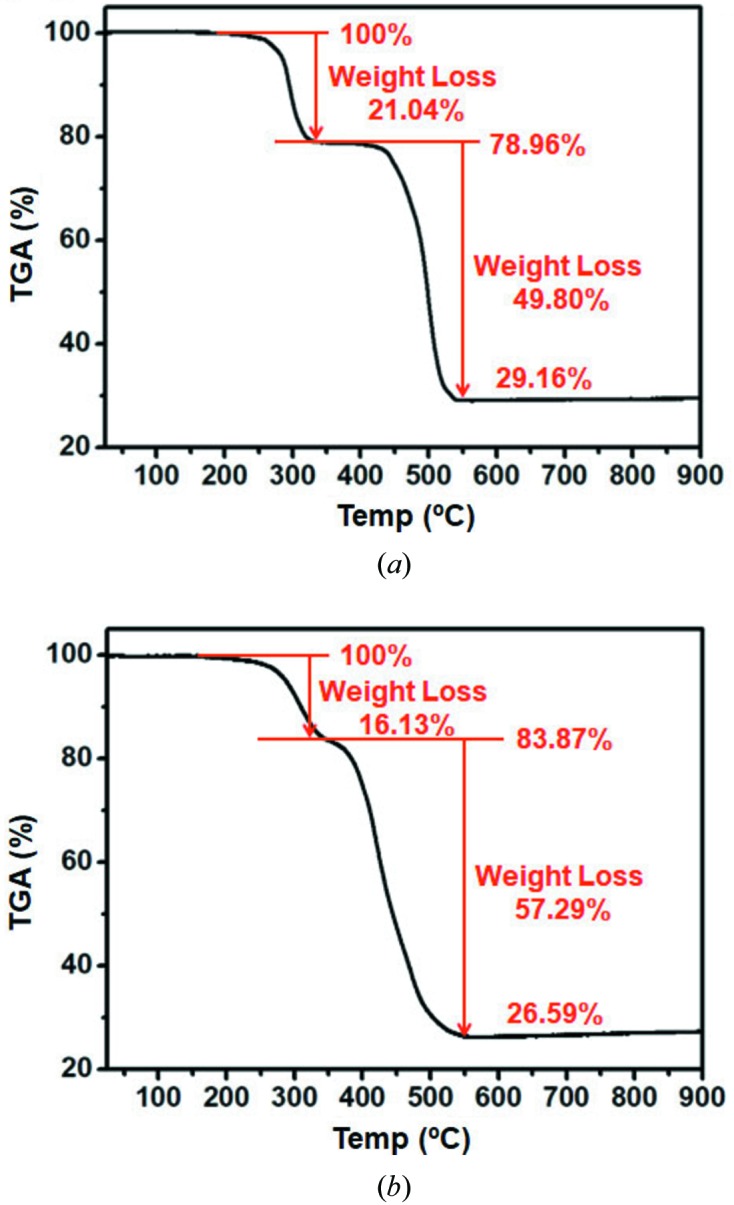
TGA curves of (*a*) the bulk rhombus of 2D-L-MOF and (*b*) the laminated disk of 2D-L-MOF.

The incorporation of PVP molecules within the laminated 2D-L-MOF was first showed using IR spectra. As shown in Fig. S6, a characteristic band from the PVP molecules at around 1657.6 cm^−1^ was detected along with the typical bands representing 2D-L-MOF. In addition, a ^1^H NMR spectrum of the laminated 2D-L-MOF digested in a mixture of DMSO-*d_6_* and acetic acid-*d_4_* indicated the incorporation of the PVP molecules within the product as the detected peaks corresponded to the PVP (Fig. S8; the sample was vigorously washed several times with DMA and methyl­ene chloride to remove undesirably involved PVP molecules). The amount of PVP incorporated within the laminated 2D-L-MOF was evaluated using the TGA curve and ^1^H NMR spectrum. First, two significant weight losses can be observed in the TGA curve of bulk 2D-L-MOF: the first weight loss (21.04%) at approximately 250°C and a second (49.80%) at approximately 450°C [Fig. 6 ▸(*a*)]. These values matched well with the theoretical weight losses corresponding to the DMA elimination [theoretical weight loss (20.19%) relating to the formula change from Zn_3_(BDC)_3_(DMA)_2_ to Zn_3_(BDC)_3_] and the decomposition of the MOF to metal oxide [theoretical weight loss (51.51%) relating to the formula change from Zn_3_(BDC)_3_ to 3ZnO], respectively. The laminated 2D-L-MOF showed a similar TGA curve [Fig. 6 ▸(*b*)], but the second weight loss occurred at a slightly low temperature compared with bulk 2D-L-MOF because of the reduced stability of the laminated 2D-L-MOF with respect to the well stacked bulk MOF. In addition, it showed different weight loss values compared with the original bulk 2D-L-MOF because of the additionally incorporated PVP molecules and the less incorporated DMA molecules with a chemical composition of [Zn_3_(BDC)_3_(DMA)_*x*_(PVP)_*y*_]_*n*_. The amounts of DMA and PVP incorporated within the laminated 2D-L-MOF were estimated using the TGA curve. The number of DMA molecules within the laminated 2D-L-MOF decreased from 2 per unit composition for the bulk MOF to *ca*. 1.7 ([Zn_3_(BDC)_3_(DMA)_1.7_(PVP)_*y*_]_*n*_) and *ca*. 0.319 mg of PVP was incorporated within 3.91 mg of the laminated 2D-L-MOF [*ca*. 8.2 wt%; Fig. 6 ▸(*b*), and see Fig. S9(*b*) for the detail calculations]. The ^1^H NMR spectrum (Fig. S8) also showed that *ca*. 1.7 DMA molecules per unit composition were involved within the laminated 2D-L-MOF. Lastly, the evaluation of the amounts of DMA and PVP in the laminated 2D-L-MOF via TGA and ^1^H NMR was fairly consistent over the several measurements.

## 4. Conclusions   

In summary, the structural and morphological control of a MOF to form a 3D cubic structure (3D-C-MOF) or a 2D layered structure (2D-L-MOF) is demonstrated by regulating the amount of reactants and the reaction conditions. A relative ratio between metal ions and organic building blocks used during the reaction is found to be critical in the selective formation of 2D-L-MOF and 3D-C-MOF, and is associated with the chemical compositions of MOFs. In addition, well shaped and uniform rhombus 2D-L-MOF microparticles are successfully synthesized in the presence of an ultrasonic dispersion. Lastly, a modified bottom-up lamination method, involving both chemical (surfactant) and physical (ultrasonication) stimuli, can laminate 2D-L-MOF layers. The synthetic strategy developed here should be a vital guideline for the selective synthesis of 2D MOFs and for the lamination of 2D MOF layers.

## Supplementary Material

Supporting figures. DOI: 10.1107/S2052252519007760/yc5019sup1.pdf
