# Supplementary material for: Competitive formation between 2D and 3D metal-organic frameworks: insights into the selective formation and lamination of a 2D MOF
Source: IUCrJ. 2019 Jun 12;6(Pt 4):681–7. doi: 10.1107/S2052252519007760 (PMC6608629; doi:10.1107/S2052252519007760)
Supplement: Supplementary file 1 [file m-06-00681-sup1.pdf]

IUCrJ

Volume 6 (2019)

Supporting information for article:

Competitive formation between 2D and 3D metal-organic frameworks (MOFs): insights on the selective formation and lamination of 2D MOF

Sojin Oh, Jeehyun Park and Moonhyun Oh

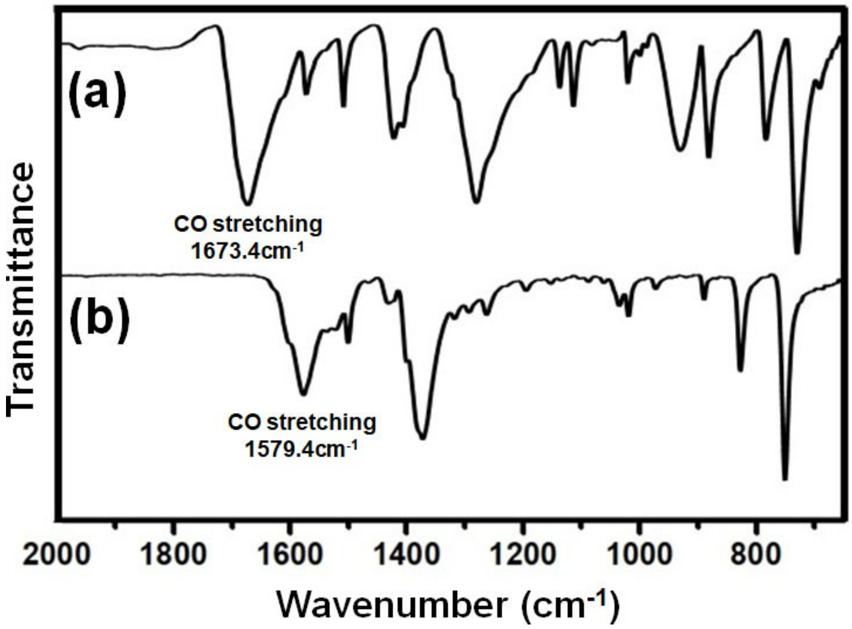

1 IR spectra of (a) H_2BDC and (b) **2D-L-MOF**.

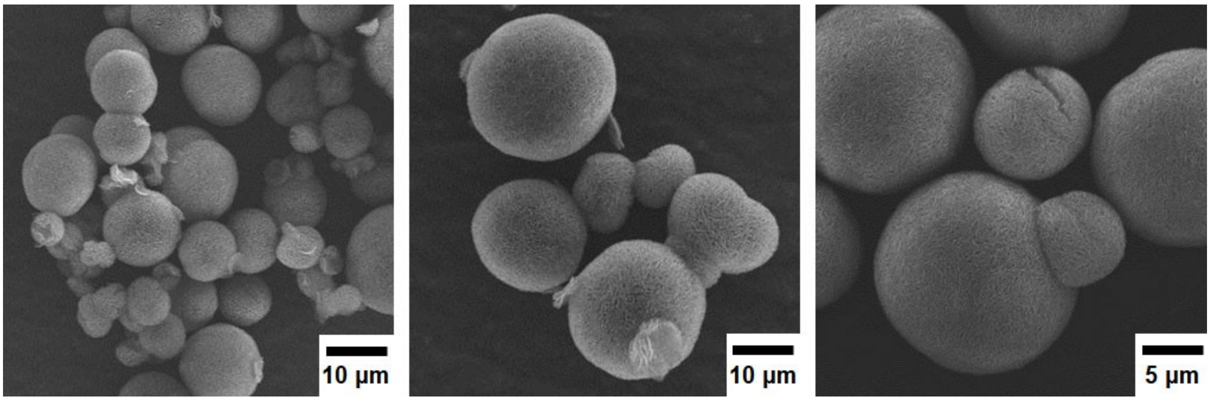

2 SEM images showing MOF product obtained from the solvothermal reaction of $Zn(NO_3)_2$ (0.54 mmol) and H_2BDC (1.62 mmol) in the presence of PVP without an ultrasonic dispersion process.

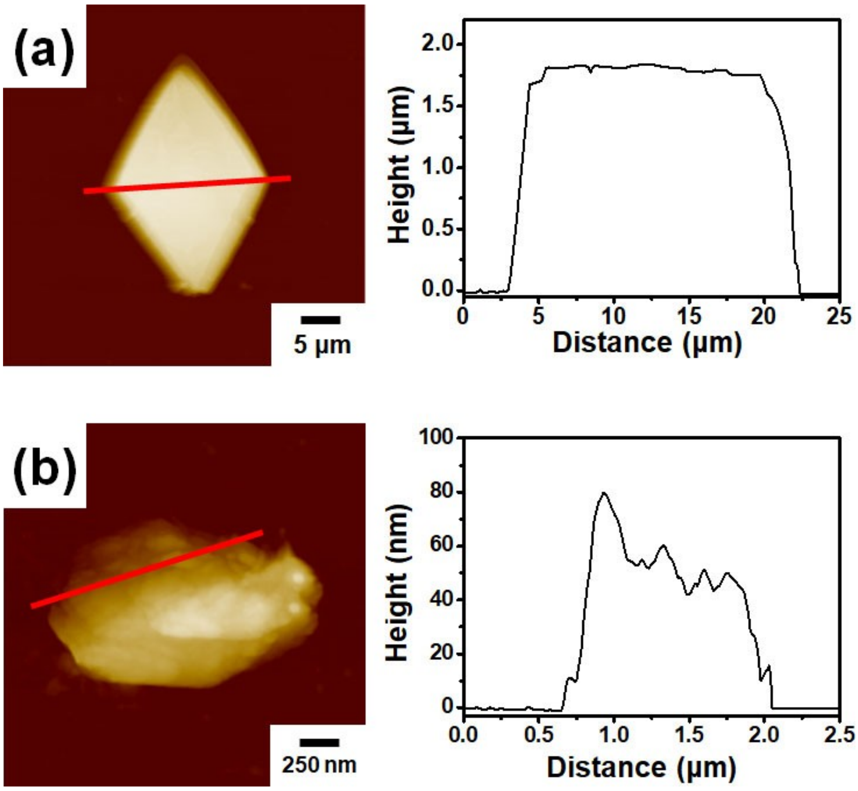

3 AFM images of (a) the rhombus **2D-L-MOF** particles obtained from the solvothermal ultrasonic reaction of $Zn(NO_3)_2$ (0.54 mmol) and H_2BDC (1.62 mmol); however, an ultrasonic dispersion process was stopped immediately after the initial seed formation and (b) the **2D-L-MOF** disks obtained from the solvothermal ultrasonic reaction of $Zn(NO_3)_2$ (0.54 mmol) and H_2BDC (1.62 mmol) in the presence of PVP. A single layer of **2D-L-MOF** was calculated to be *ca.* 1.06 nm based upon its single crystal structure. However, the thickness of laminated **2D-L-MOF** and the precise information on the number of layers could not obtain due to its waved-morphology. There are *ca*. ten layers of **2D-L-MOF** in a thin area presenting at the outside of particle shown in (b).

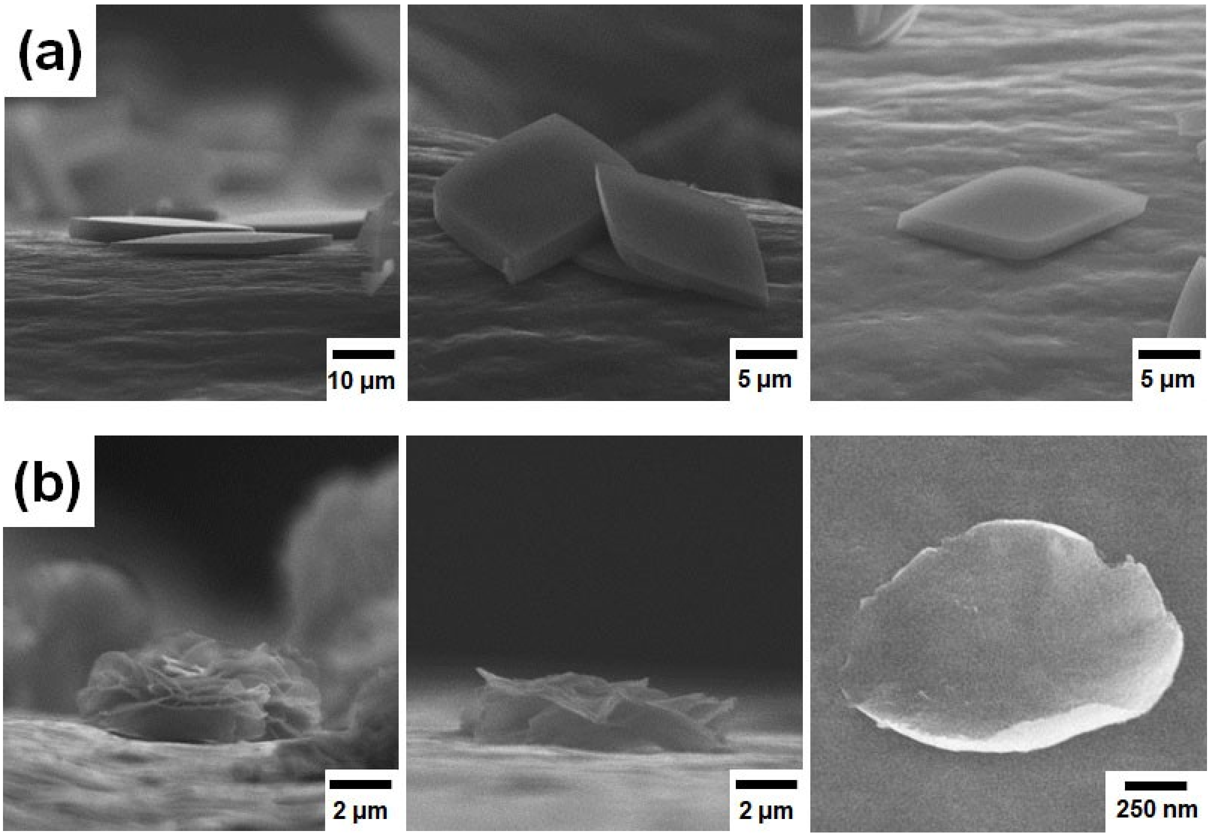

4 SEM images of samples measured at the side view. (a) The rhombus **2D-L-MOF** particles obtained from the solvothermal ultrasonic reaction of $Zn(NO_3)_2$ (0.54 mmol) and H_2BDC (1.62 mmol); however, an ultrasonic dispersion process was stopped immediately after the initial seed formation and (b) the **2D-L-MOF** disks obtained from the solvothermal ultrasonic reaction of $Zn(NO_3)_2$ (0.54 mmol) and H_2BDC (1.62 mmol) in the presence of PVP. In special, SEM images in (b) revealed its waved-morphology.

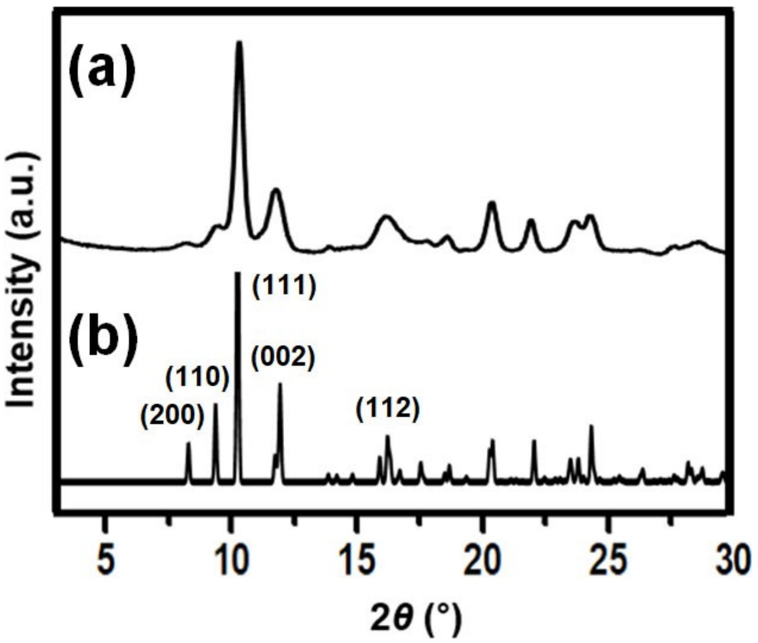

5 (a) PXRD pattern of the laminated **2D-L-MOF** disks obtained from the solvothermal ultrasonic reaction of $Zn(NO_3)_2$ and H_2BDC in the presence of PVP. (b) Simulated PXRD pattern of **2D-L-MOF**.

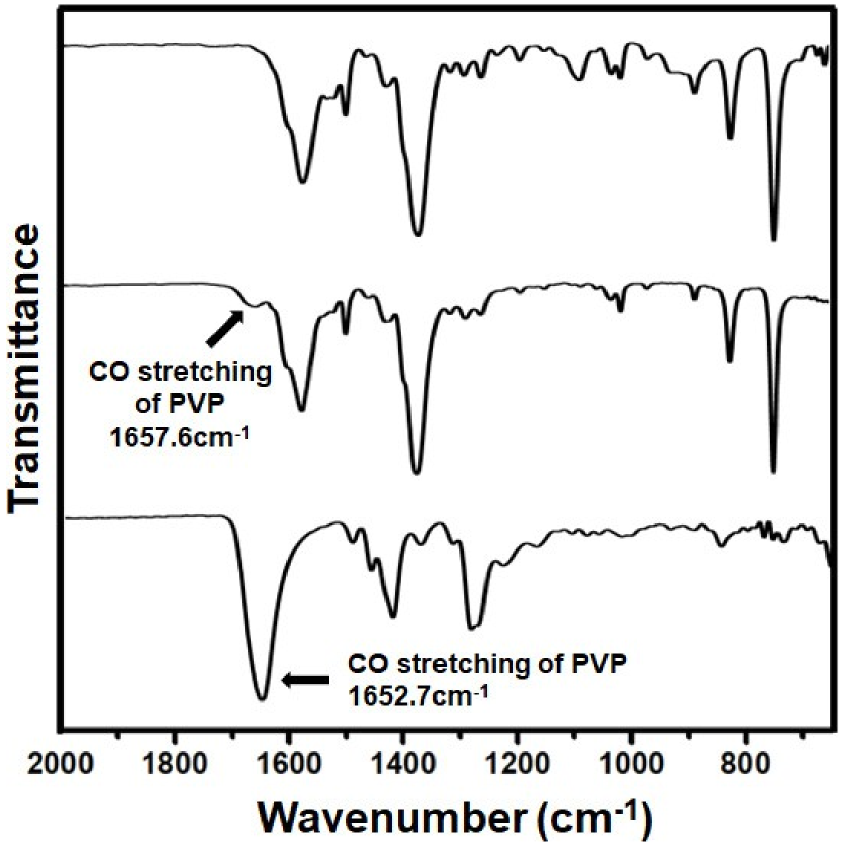

6 IR spectrum of the laminated **2D-L-MOF** disks (middle) obtained from the solvothermal ultrasonic reactions of $Zn(NO_3)_2$ and H_2BDC in the presence of PVP. For comparison, IR spectra of the bulk **2D-L-MOF** rhombus particles (top) and PVP (bottom) are included.

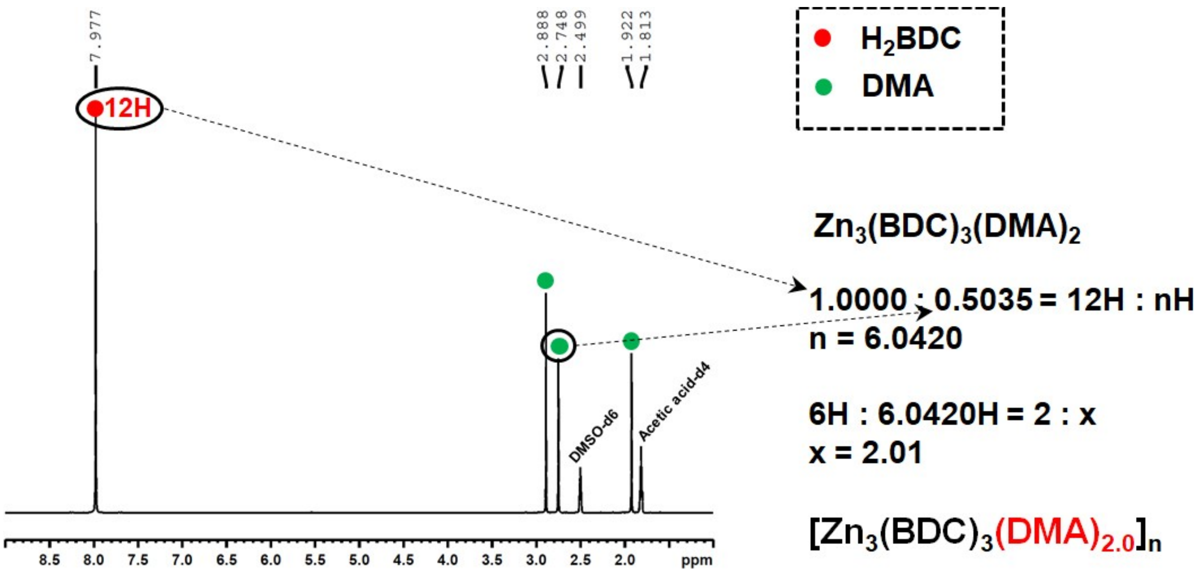

7 ^{1}H NMR spectrum of the bulk **2D-L-MOF** rhombus particles obtained from the solvothermal ultrasonic reaction of Zn^{2+} and H_2BDC. The number of DMA molecules within the bulk **2D-L-MOF** was calculated at ca. 2.0 per unit composition $[Zn_3(BDC)_3(DMA)_{2.0}]_n$ based upon ^{1}H NMR spectrum.

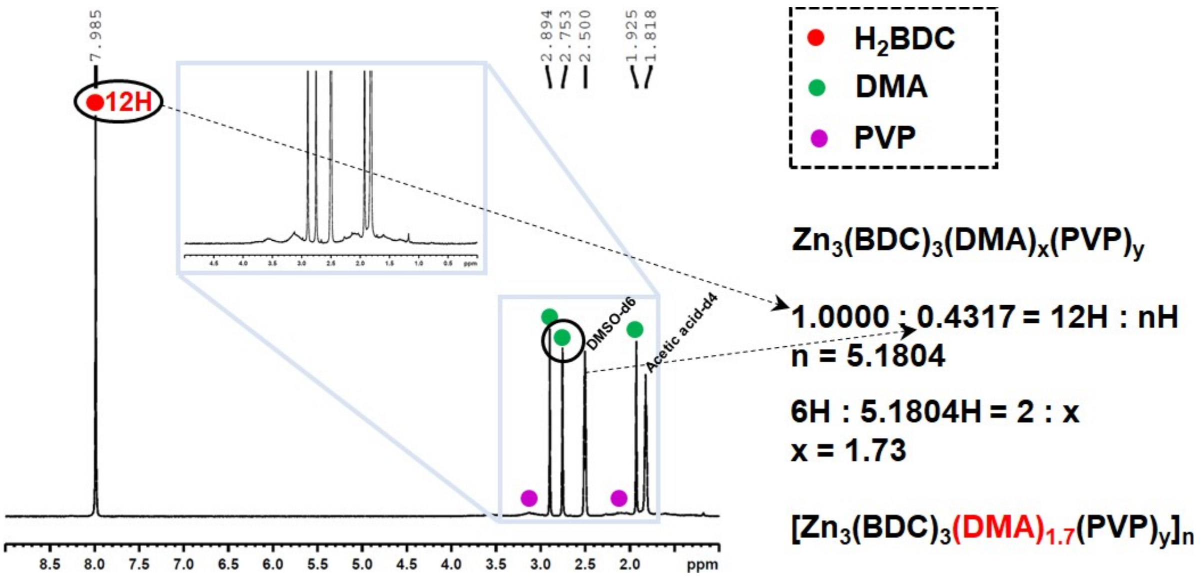

8 ^{1}H NMR spectrum of the laminated **2D-L-MOF** disks obtained from the solvothermal ultrasonic reaction of Zn^{2+} and H_2BDC in the presence of PVP. The number of DMA molecules within the laminated **2D-L-MOF** was calculated at ca. 1.7 per unit composition $[Zn_3(BDC)_3(DMA)_{1.7}(PVP)_y]_n$ based upon ^{1}H NMR spectrum.

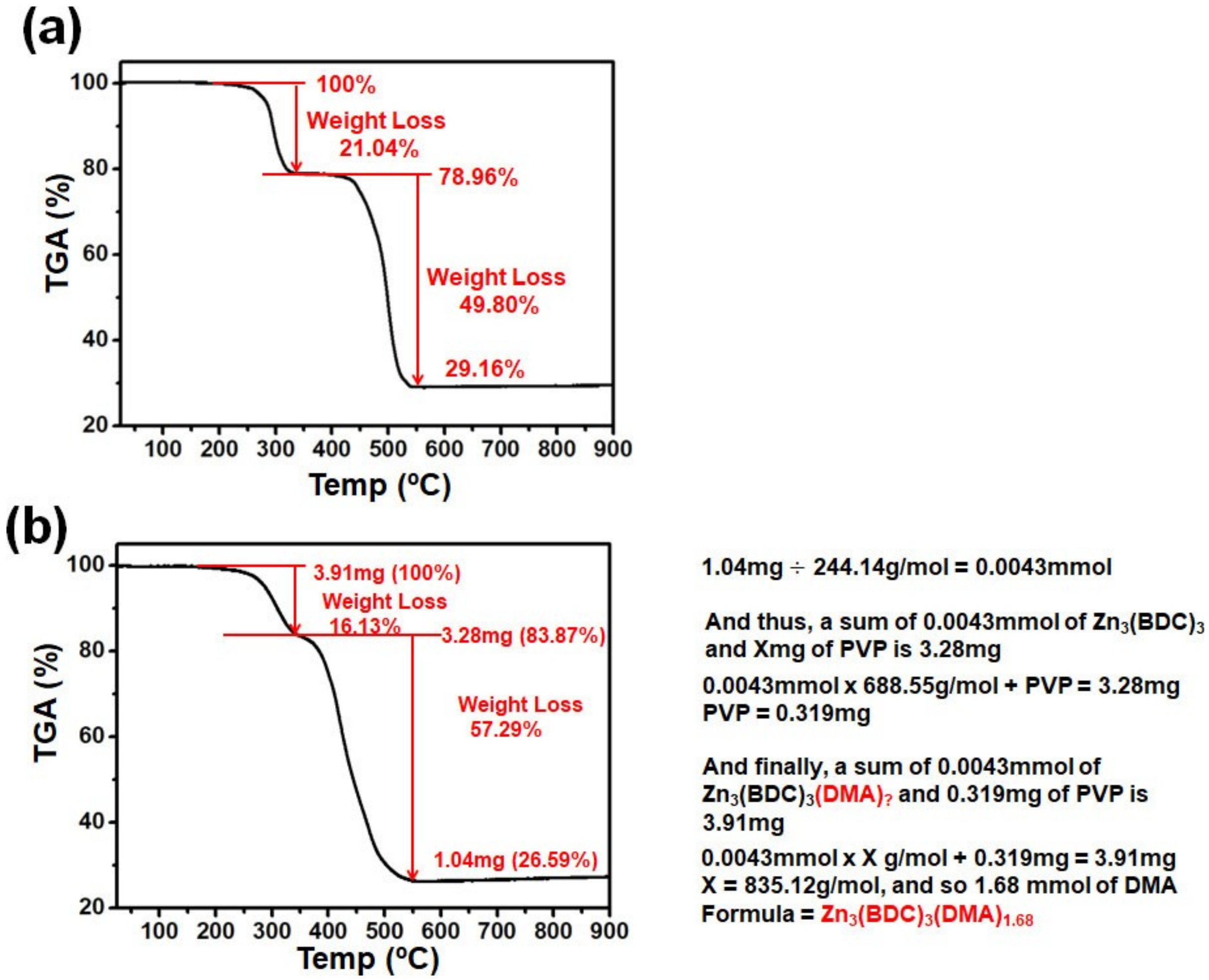

9 TGA curves of (a) the bulk **2D-L-MOF** rhombus particles and (b) the laminated **2D-L-MOF** disks. The amounts of DMA and PVP incorporated within the laminated **2D-L-MOF** were calculated based upon TGA curve.
